# Fruitful analysis of sex chromosomes reveals X-treme genetic diversity

**DOI:** 10.1186/s13059-016-1115-9

**Published:** 2016-11-29

**Authors:** Angela M. Taravella, Melissa A. Wilson Sayres

**Affiliations:** 1 School of Life Sciences, Arizona State University, Tempe, AZ 85281 USA; 2 Center for Evolution and Medicine, The Biodesign Institute, Arizona State University, Tempe, AZ 85281 USA

## Abstract

A new study on sex chromosome evolution in papaya helps to illuminate sex chromosome biology, including deviations from expected trajectories.

Please see related Research article: https://genomebiology.biomedcentral.com/articles/10.1186/s13059-016-1095-9

## Introduction

**Fig. 1:**
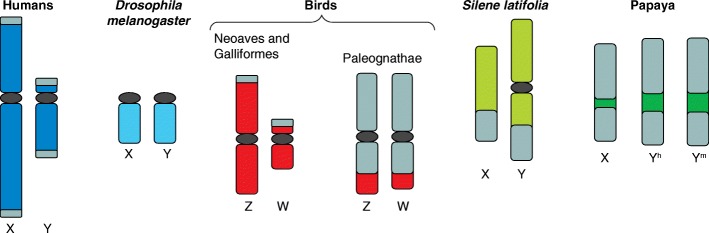
Sex chromosomes across taxa reveal variation in sex-linked morphology. A snapshot of the current understanding of morphological variation in sex chromosomes across diverse taxa shows how incorporating studies of young sex chromosomes provides a new perspective. The colored regions represent regions that do not recombine between the two sex chromosomes, whereas the grey color represents regions that do recombine, also known as pseudoautosomal regions (PARs). Although not exact, the approximate relative sizes of the sex chromosomes are represented. Human sex chromosomes have two small PARs at each tip. The fruit fly *Drosophila melanogaster* does not have recombination in males, thus there are no PARs on its sex chromosomes. Initial studies of birds in the Neoaves and Galliforms clades suggested that their sex chromosomes, despite being heteromorphic in females (ZW), mimicked the evolution of human and *Drosophila* male heteromorphic (XY) sex chromosomes, exhibiting highly degraded W chromosomes. However, further work in Paleognath birds, such as the emu, revealed that not all old sex chromosome systems will have a degenerated heteromorphic sex chromosome (W or Y). In contrast to birds, mammals and flies, the plants studied to date have much younger sex chromosomes, which facilitate the study of how quickly recombination suppression evolves between the sex chromosomes. The 10–20 million year old X and Y chromosomes of *Silene latifolia* have already experienced three recombination-suppression events, but there are small regions on the distal arm of these sex chromosomes that can still recombine. The evolutionary rate at which quick recombination suppression occurs may, however, be highly variable. The 7-million-year-old papaya sex chromosomes are largely able to recombine, with relatively small sex-specific regions. Curiously, in both papaya and *S. latifolia*, the Y-specific regions are larger than the X-specific regions. It is only by studying diverse taxa that we can develop truly general expectations for sex chromosome evolution

The evolution of sex chromosomes has occurred many times in highly divergent taxa. The bulk of the research that has shaped our expectations for their evolutionary path has focused on model organisms that have older sex chromosomes; in these organisms, the two sex chromosomes are highly differentiated from each other in form and function (Fig. 1; [1]). By contrast, many plant species have young sex chromosomes that are still largely homologous; these chromosomes can provide information about the initial stages of sex chromosome evolution. Neutral expectations of genetic diversity predict that autosomes will have the highest genetic diversity, followed by the X (or Z) chromosome, with the Y (or W) chromosome having the least diversity (reviewed in [2]). A recent study by VanBuren et al. [3] published in *Genome Biology* revealed an unexpected trend in genetic diversity in wild and domestic papaya populations: X-linked loci harbor the lowest levels of diversity within the genome, ten-fold lower than autosomal diversity and twelve-fold lower than Y-linked diversity. The authors propose that this pattern of diversity is due to a strong selective sweep in wild papayas that occurred prior to domestication.

## Young versus old sex chromosomes

Ancient sex chromosomes, such as those independently derived in mammals, birds, *Drosophila*, and *Caenorhabditis elegans*, can provide information about the evolutionary fate of sex chromosomes. Typically, in these systems, recombination suppression between homologous chromosomes results in degeneration and reduced diversity on the heteromorphic sex chromosome [4], as has been observed in humans [5]. However, these trends are not consistently observed. For example, even though all birds’ sex chromosomes are homologous (the Z-W pair derived from the same shared ancestral autosomal pair about 120 million years ago), the Z and W chromosomes of ratite birds are largely undifferentiated, whereas other bird taxa have highly differentiated sex chromosomes (Fig. 1; [6]).

Organisms that have old sex chromosomes provide fascinating information about the long-term consequences of sex-linkage and the expected evolutionary trajectories of sex chromosomes, yet shed little light onto the early stages of sex chromosome evolution. For example, although studies of the current human pseudoautosomal regions (PARs) can shed light on current patterns of X-Y recombination [7], studies of the young (8–20 million years old) sex chromosomes of the flowering plant *Silene latifolia* have elucidated how recombination suppression spreads early in the process of differentiation [8]. Young sex chromosomes are ideal for studying recombination suppression because they generally tend to have large PAR regions and are still actively evolving recombination suppression between the sex chromosomes. Initial data suggest that young sex chromosomes may be very active; there is evidence that the *S. latifolia* PAR has experienced at least two additions and multiple recombination suppression events in just 5 million years, whereas the human PAR has been stable for approximately 30 million years [8].

## Neutral expectations for genetic diversity across the genome

Mutation rate, selection, and effective population size (N_e_) together affect observed genetic diversity. Under neutral scenarios with equal sex ratios, a N_e_ ratio 4:3:1 of autosomes, X chromosome, and Y chromosome should occur; the reasoning behind this ratio is that for every genetic female (XX), there will be one genetic male (XY), and for this pair, we expect representation of four autosomes, three X chromosomes, and one Y chromosome [2]. Consistent with these expectations, diversity is reduced in the Y-linked regions in *S. latifolia* relative to X-linked regions [9]. However, deviations from this ratio are common, and can be explained by a variety of evolutionary mechanisms. Population structure can increase genetic diversity in the Y chromosome because migration is either reduced or absent, and alleles can fix faster, independently, due to genetic drift [3]. Diversity can be reduced at selected and linked regions due to positive selection and selective sweeps, or by purifying selection and background selection; the effect of linked selection is stronger for regions without recombination [4, 5]. Alternatively, diversity on the sex chromosomes can be affected by sex-biased variance in reproductive success, and sex-biased introgression (reviewed in [2]). Comparisons of diversity among the autosomes and sex chromosomes are needed to disentangle the relative forces affecting diversity in each region.

## Surprising patterns of genetic diversity in papaya

Wild papaya (*Carica papaya*) is dioecious, with separate male and female plants; by contrast, cultivated papaya has only females and hermaphrodites. The divergence of male (Y^m^) and hermaphrodite (Y^h^) chromosomes in papaya occurred recently, ~4000 years ago, probably as a result of papaya domestication and the origin of agriculture in Mesoamerica [10]. In their recent study, VanBuren et al. [3] analyzed population structure and diversity in the X-linked, Y-linked, pseudoautosomal, and autosomal regions of the papaya genome in both wild populations in Costa Rica and cultivated varieties, reporting very unexpected patterns.

In contrast to the findings of previous studies of sex chromosome diversity, the region with the highest diversity is the Y chromosome in both wild and cultivated papayas [3]. Further, for wild papaya, genetic diversity, measured as the average number of pairwise differences per site (π), was lowest for X-linked loci at 0.00038, intermediate for autosomal (π = 0.0017) and pseudoautosomal (π = 0.0020) loci, and highest for Y-linked loci (π = 0.0021); X-linked π in the cultivated samples was reported to be about half the value of that in the wild samples, probably because of a population bottleneck during domestication [3]. By comparing variation at autosomal and X-linked synonymous sites, VanBuren et al. show that an unrealistic mutation rate would be needed to explain the low observed X-linked diversity, thus excluding difference in mutation rate as a primary explanation for the reduced diversity.

VanBuren et al. then conducted a series of tests to infer the processes that may have led to the extreme low genetic diversity observed for X-linked genes. Tajima’s D calculations for the X-linked region and PAR for cultivated samples suggest a genome-wide bottleneck during domestication that involved selection for hermaphroditism (which is inherited via a locus on the Y^h^ chromosome). The lack of recombination in Y-specific regions has probably led to population differentiation and genetic drift between wild and domesticated species that resulted in the high Y-linked diversity. Curiously, the researchers observed little to no population structure on the X chromosome, and low fixation index (F_st_) between wild and domesticated papayas on the X chromosome, suggesting ongoing gene flow between domesticated and wild populations. Additionally, there was little evidence of gene flow in the PAR regions. On the basis of the extremely low sequence diversity of the X-linked sequences and the lack of population structure, the authors propose that genetic hitchhiking may have reduced diversity on the X chromosome, suggesting a recent selective sweep caused by a beneficial trait spreading prior to domestication. The genes that were involved in this sweep and sex-determining genes remain unknown, and open for investigation.

## Expect the unexpected

When it comes to sex-linked diversity, the null hypothesis of neutral equilibrium expectations are rarely observed. However, typical deviations from this null occur so often in one direction (significantly reduced Y or W diversity) that they can become de facto expectations. This new work on papaya sex-linked diversity reminds us of the myriad ways in which evolutionary forces shape genomic variation, and even sometimes surprise us.

## Abbreviation

PAR: Pseudoautosomal region

## References

[1] Bachtrog D, Mank JE, Peichel CL, Kirkpatrick M, Otto SP, Ashman T (2014). Sex determination: why so many ways of doing it?. PLoS Biol.

[2] Webster TH, Wilson Sayres MA (2016). Genomic signatures of sex-biased demography: progress and prospects. Curr Opin Genet Dev.

[3] VanBuren R, Wai CM, Zhang J, Han J, Arro J, Lin Z, et al. Extremely low nucleotide diversity in the X-linked region of papaya caused by a strong selective sweep. Genome Biol. 2016. doi: 10.1186/s13059-016-1095-9. 10.1186/s13059-016-1095-9 PMC5125041 27890017

[4] Charlesworth B, Charlesworth D (2000). The degeneration of Y chromosomes. Philos Trans R Soc Lond B.

[5] Wilson Sayres MA, Lohmueller KE, Nielsen R (2014). Natural selection reduced diversity on human Y chromosomes. PLoS Genet.

[6] Vicoso B, Kaiser VB, Bachtrog D (2013). Sex biased gene expression at homomorphic sex chromosomes in emus and its implication for sex chromosome evolution. Proc Natl Acad Sci U S A.

[7] Cotter DC, Brotman SM, Wilson Sayres MA (2016). Genetic diversity on the human X chromosome does not support a strict pseudoautosomal boundary. Genetics.

[8] Bergero R, Qiu S, Forrest A, Borthwick H, Charlesworth D (2013). Expansion of the pseudo-autosomal region and ongoing recombination suppression in the *Silene latifolia* sex chromosomes. Genetics.

[9] Qiu S, Bergero R, Forrest A, Kaiser VB, Charlesworth D (2010). Nucleotide diversity in *Silene latifolia* autosomal and sex-linked genes. Proc R Soc B.

[10] VanBuren R, Zeng F, Chen C, Zhang J, Wai CM, Han J (2015). Origin and domestication of papaya Y^h^ chromosome. Genome Res.

